# Transitioning from Unilateral to Bilateral Upper Limb Tremor Therapy for Parkinson’s Disease and Essential Tremor Using Botulinum Toxin: Case Series

**DOI:** 10.3390/toxins10100394

**Published:** 2018-09-27

**Authors:** Olivia Samotus, Jack Lee, Mandar Jog

**Affiliations:** 1 Department of Clinical Neurological Sciences, London Health Sciences Centre—Lawson Health Research Institute, 339 Windermere Road, A10-026, London, ON N6A 5A5, Canada; osamotus@uwo.ca (O.S.); jack.lee@lhsc.on.ca (J.L.); 2 Schulich School of Medicine and Dentistry, University of Western, 1151 Richmond Street, London, ON N6A 3K7, Canada

**Keywords:** Parkinson’s disease, essential tremor, tremor, movement disorders, Botulinum toxin, kinematics, upper limb biomechanics, joint biomechanics, diagnostic guidance, clinical decision support

## Abstract

Botulinum toxin type A (BoNT-A) injections guided by kinematic analysis for unilateral upper limb essential tremor (ET) and Parkinson’s disease (PD) tremor therapy has demonstrated efficacy, improvements in quality of life (QoL) and arm functionality. In this open-label pilot trial, 5 ET and 2 PD participants decided to switch from receiving long-term unilateral arm treatment to now bilateral BoNT-A arm therapy in their other tremulous arm which worsened over time. Injection patterns were based on kinematic analysis. Efficacy endpoints including kinematic analysis, Fahn-Tolosa-Marin tremor rating scale, QoL questionnaire, and maximal grip strength were collected over 2 treatments and 2 follow-up visits totaling 18-weeks. BoNT-A decreased wrist tremor amplitude by 84.6% and 89.6% 6-weeks following the 1st injection in the newly-treated limb in ET and PD participants, respectively. PD participants started with worse QoL but demonstrated an additional improvement in QoL by 29.9% for switching to bilateral treatment, whereas ET participants did not. Left and right arm tremor also did not share commonalities in severity or dose. This preliminary finding suggests trends for transitioning to bilateral therapy and warrants further studies to evaluate efficacy of bilateral tremor BoNT-A therapy in a larger cohort of PD and ET patients.

## 1. Introduction

One-third of Parkinson’s disease (PD) patients experience tremor-related quality of life (QoL) problems that interfere with daily activities, such as eating and dressing, which induce psychosocial stress in more than 25% of patients [1]. Tremor is a functional interference and social embarrassment for those with essential tremor (ET) and hence many seek therapy. ET and PD tremor amplitudes are generally asymmetric in severity and frequency [2,3] however it is typical to observe worsening of PD tremor severity over the disease progression where ET severity increase is correlated to patient age and duration of tremor and thus severity gradually worsens with time [4]. Traditional oral medications, such as levodopa for PD tremor and beta-blockers and anticonvulsants for ET, provide suboptimal benefit although are frequently coupled with significant adverse events [5,6,7] and many of these patients stop their oral treatments within the first year [8,9].

Visually assessed, targeted therapy by botulinum toxin type A (BoNT-A) injections for one arm have been shown to produce reasonable reduction of ET and PD wrist tremor severities although significant muscle weakness and limited functional benefit has warranted many patients to discontinue treatment [10,11,12,13,14]. Samotus et al. have demonstrated the importance of utilizing objective tremor analysis by measuring the severity and direction of tremor along the whole arm using motion sensor devices to guide the clinical determination of BoNT-A injection muscle groups to treat, thereby limiting the likelihood of muscle weakness over serial treatments [15,16,17]. Furthermore, a significant reduction in ET and PD tremor has been shown when treating the side of the arm with the most bothersome upper limb following the first treatment, which was sustained by a 76% and 70% improvement, respectively, up to 96 weeks compared to baseline tremor severity measures [17]. In addition, ET and PD patients experience improvement in arm function as functional disability caused by tremor was significantly reduced by 46.3% and 31.3%, respectively [17]. While ET participants showed a 46.1% improvement in QoL when treated in their most disabling limb, PD participants demonstrated no change in QoL [17].

This is the first proof of principle study to date to demonstrate that ET and PD participants who already received beneficial unilateral BoNT-A therapy can transition to bilateral BoNT-A injections for their upper limb tremor for further improvement. The decision to transition was based upon each participant’s discretion and muscle injection patterns were based on kinematic tremor analysis.

## 2. Results

### 2.1. Study Population

A total of 7 (5 ET and 2 PD) participants who were no longer satisfied with their BoNT-A treatment chose to transition from unilateral to bilateral upper limb injections as they felt tremor in their untreated arm to be bothersome. These participants were part of the same cohort of individuals published in previous tremor papers from the London Movement Disorders Centre [15,16,17]. Clinical scores, demographics, number of injection cycles and the injection parameters in the first treated limb of the 7 participants are summarized in Table 1. All participants were right-handed and initially received BoNT-A therapy in their most bothersome tremor arm which was the right arm; a mean of 6 ± 3 additional injection cycles were administered following the 96-week study [17] before transitioning to bilateral therapy (initiation of treatment in their left arm). All participants had greater tremor severity in their original treated (right) arm at the start of the 96-week study. Three ET participants received monotherapy of BoNT-A and were not on any other tremor related medication.

**Figure 1:**
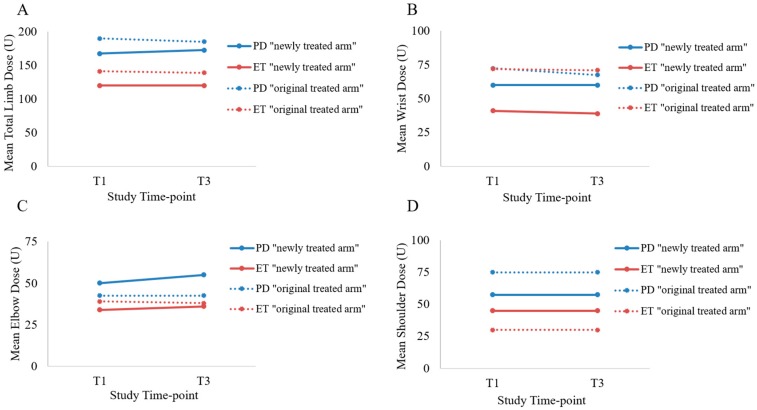
Mean BoNT-A dosages in the whole arm (**A**), wrist (**B**), elbow (**C**), and shoulder (**D**) joints are plotted for each limb for Parkinson’s disease (PD) and essential tremor (ET) participants.

The mean number of muscles and the dosages allocated to elbow and shoulder muscle groups were similar between both upper limbs for all participants. However, the mean wrist dose for the newly treated (left) arm was 46.4 ± 17.3 U compared to the optimized mean wrist dose of 72.1 ± 36.2 U in the right arm for all participants (Supplementary Tables S1 and S2). For all transition participants, a similar mean total dose was administered for the two treatment cycles in the left arm, 133.6 ± 47.3 U and 135.0 ± 48.0 U at the 1st injection (T1) and 2nd injection (T3), respectively (Figure 1). Mean BoNT-A dosages in the wrist muscles were lower in the newly treated arm (8.4 ± 4.9 U) compared to the original treated arm (10.8 ± 6.3 U) (Supplementary Tables S3 and S4). Dosages allocated to the elbow and shoulder muscles were also lower in the newly treated arm (16.6 ± 8.7 U) compared to the original treated arm (23.0 ± 5.8 U).

### 2.2. Fahn-Tolosa-Marin (FTM) Tremor Rating and Arm Functionality

**Figure 2:**
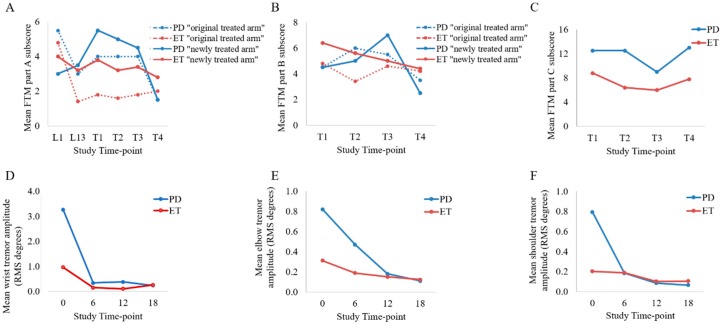
Mean Fahn-Tolosa-Marin (FTM) sub-scores for part A (**A**), part B (**B**), and part (**C**) and mean root mean square (RMS) tremor amplitudes at the wrist (**D**), elbow (**E**) and shoulder (**F**) joints for PD and ET participants across a period of 18 weeks. T1 and T3 study visits were injection visits 12-weeks apart and T2 and T4 were follow-up visits 6-weeks following an injection. For tremor severity (FTM part A), time-points at L1 and L13 indicating week 0 and week 96, respectively, of the previously published unilateral tremor-BoNT-A treatment study were included.

Prior to treating the left arm (untreated), ET tremor severity was similar from L1 (week 0 of the original unilateral 96-week study [17]) to first transition visit (T1), indicating no increase in left arm tremor amplitude (Figure 2A). As for the PD participants’ newly treated (left) arm, tremor severity (FTM part A) worsened by 83.3% (mean + 1.5 point difference) from week 0 (L1) to transition initiation (T1).

Following the second treatment (T4) in the newly treated (left) arm, a mean 26.3% tremor reduction (−1.0 FTM part A point difference) from mild-moderate (3.8 ± 1.8) at pre-treatment (T1) to slight-mild (2.8 ± 1.6) tremor severity was observed in the ET group (Figure 2A). A 31.3% (mean −2.0 point difference) improvement in fine motor tasks (FTM part B) was observed at T4 (2.5 ± 2.3) compared to T1 (4.5 ± 2.3) (Figure 2B). In the previously treated and optimized (right) limb, tremor severity and fine motor skills did not change over the two treatment cycles (Figure 2A,B). A reported 31.8% (mean −2.8 point difference) and a 11.4% (mean −1.0 point difference) improvement in FTM part C scores were observed at time of re-injection (T3; 6.0 ± 3.2) and following the 2nd injection (T4; 7.8 ± 4.3), respectively, compared to pre-injection (T1; 8.8 ± 5.6) demonstrating a trending improvement in daily activities for ET participants (Figure 2C).

In the PD group, a 72.7% reduction (mean −4.0 point difference) in tremor severity was observed from 5.5 ± 1.9 points at T1 to 1.5 ± 1.6 points at T4 demonstrating a reduction in severity from mild-moderate to a slight tremor severity (Figure 2A). A 44.4% improvement (mean −2.0 point difference) in fine motor skills (FTM part B) was observed from 4.5 ± 2.3 points at T1 to 2.5 ± 2.3 points at T4 in the PD group (Figure 2B). Functional disability caused by tremor (FTM part C) was reduced by 28.0% (mean −3.5 point difference) from 12.5 ± 5.6 points at T1 to 9.0 ± 3.2 points at time of re-injection (T3) (Figure 2C).

### 2.3. Kinematic Tremor Analysis

Four (1 PD and 3 ET) and two (1 PD and 1 ET) participants experienced their most severe wrist tremor amplitude during “posture” and “load” tasks, respectively, in the newly treated (left) arm as compared to their original treated (right) arm which was pre-dominantly most severe during the “load” tasks (Table 2). Similarly, five (2 PD and 3 ET) participants experienced their highest elbow tremor during “posture” in the newly treated arm while majority of the elbow tremor in the original treated arm was captured during “load” tasks. For shoulder tremor, the original treated arm most severe tremor was captured during “load” tasks, while for the newly treated arm there was no obvious task specific trend.

Prior to the treatment in the newly treated (left) arm, the PD participants (3.3 ± 1.9 RMS-degrees) exhibited a higher wrist tremor severity as compared to the ET group (1.0 ± 0.9 RMS-degrees). For ET participants in the newly treated arm, mean wrist RMS tremor amplitude was reduced by 84.6% (mean −0.8 RMS-degree difference) from 1.0 ± 0.9 RMS-degree at T1 to 0.1 ± 0.1 RMS-degree following the 1st injection (T2) and was maintained by a tremor reduction of 73.4% (0.2 ± 0.3 RMS-degrees) at following the 2nd injection (T4) (Figure 2D). Mean elbow and shoulder tremor amplitudes were reduced by 60.2% and 47.5%, respectively, following the 2nd injection (T4) as compared to pre-injection (T1) (Figure 2E,F). Similar changes in tremor severity were observed in the two PD participants, a mean reduction of 92.6%, 86.6% and 91.8% in wrist, elbow and shoulder tremor amplitude was observed following the 2nd injection compared to T1 (Figure 2D,F).

### 2.4. Quality of Life Measures and Safety Outcomes

**Figure 3:**
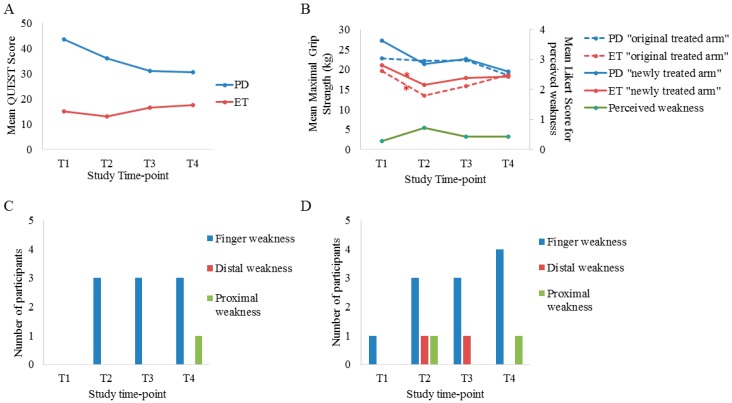
Mean quality of life for essential tremor questionnaire (QUEST) scores (**A**), changes in mean maximal grip strength and participant-perceived muscle weakness (**B**), and number of participants with a manual muscle testing (MMT), rating ≤ 3 in the newly treated arm (**C**) and in the original treated arm (**D**) across the transition 18-week period involving two BoNT-A treatments at T1 and T3 were plotted. Tremor impact on QoL was reduced demonstrated by a reduced QUEST score.

For the ET cohort that had bilateral tremor with predominant severity originally in the treated right arm, the transition into bilateral tremor treatment resulted in an additional improvement in QoL score (QUEST) by 13.3% (mean −2.0 point difference) from 15.0 ± 10.1 at time of 1st bilateral treatment (T1) to 13.0 ± 9.4 6-weeks following the 1st injection (T2), although a 17.3% (mean +2.6 point difference) worsening in QoL was observed following the 2nd injection at T4 (17.6 ± 13.8) (Figure 3A). For the PD participants, QoL was improved by 29.9% (mean −13.0 point difference) from 43.5 ± 4.9 at T1 to 30.5 ± 4.9 QUEST score at T4.

In the ET population, the mean maximal grip strength in the newly treated arm was reduced by 23.1% (mean −4.9 kg difference) from 21.1 ± 9.1 kg at T1 to 16.2 ± 9.2 kg following the 1st injection at T2, however this reduction in maximum grip strength was not noticeable at time of re-injection at T3 (mean −3.1 kg difference; 17.9 ± 9.1 kg) or after at T4 (mean −2.8 kg difference; 18.3 ± 10.7 kg) (Figure 3B). In the original treated arm, which had received serial treatments prior to bilateral treatment, a reduction in mean grip strength by 31.4% (mean −6.2 kg difference) from 19.7 ± 7.1 kg at T1 to 13.5 ± 9.0 kg at T2, was observed in ET. In the PD group, the mean maximal grip strength in the newly treated arm was reduced by 21.5% (mean −5.8 kg difference) from 27.2 ± 14.8 kg at T1 to 21.3 ± 13.7 kg following the first injection at T2 and was further reduced to 19.5 ± 12.5 kg following the second injection at T4. In the original treated arm, mean maximal grip strength was similar from T1 to T3 although following the 2nd injection at T4, a 19.0% reduction in mean grip strength (−4.3 kg difference) was observed compared to 22.8 ± 12.0 kg at T1.

When questioning participants of their perceived change in hand and arm strength, the mean Likert score for participant-perceived weakness reported only slight perceived weakness in injected muscles following the 1st injection (T2) (Figure 3B). At the re-injection visit (T3), perceived muscle weakness was scored as none to slight mild weakness 6-weeks following the 2nd injection (T4).

Based on MMT of the newly treated (left) arm, three participants (1 PD and 2 ET) experienced finger weakness following the first treatment, at re-injection and following the second treatment (T2–T4) (Figure 3C). An ET participant was felt to have proximal (triceps) weakness following the second treatment (T4). In the original treated arm, 3 participants (1 PD and 2 ET) experienced finger weakness during the first treatment cycle (T2–T3) and 4 participants (1 PD and 3 ET) experienced middle finger weakness following the second treatment (T4) (Figure 3D). An ET participant experienced wrist flexor/extensor weakness at T2 and T3 and an ET participant experienced triceps weakness at T4.

## 3. Discussion

In this open-label, pilot, clinical phase II study, 5 ET and 2 PD participants chose to switch to bilateral tremor treatment as they perceived their untreated left arm to be now bothersome enough to warrant treatment. Prior to bilateral treatment, ET participants had been treated for their right arm tremor and already experienced significant functional and quality of life (QoL) improvement in their arm function as reported by all FTM and QUEST scores with sustained benefit over 96 weeks [17]. Once bilateral treatments were introduced, the tremor severity was vastly reduced at the wrist, elbow, and shoulder in the left arm. FTM functional scale reported an additional mild improvement, however scores for QoL did not further improve (Figure 2A and Figure 3A). Given that these 5 ET participants prior to any BoNT-A therapy had asymmetric bilateral tremor (tremor was worse on right side), the participants still desired treatment to possibly hide and suppress the visual cues of their left arm tremor which had become more prominent in comparison to their treated right arm.

For the 2 PD participants in the newly treated (left) arm, tremor severity also was vastly reduced (Figure 2C). At the start of bilateral treatment, the two PD participants had worse QoL (QUEST) scores as compared to their ET counterparts, which may explain the large additional improvement in QoL (29.9% increase) following two treatment cycles (Figure 3A). Likewise, the PD participants had a measurable worsening of their left arm tremor leading up to the bilateral treatment, while this was not the case for the ET group who perceived their left arm to be more noticeable in tremor severity. Thus, the decision of the 2 PD participants to receive bilateral therapy could be due to their priority to further improve arm function and overall quality of life, and less likely due to tremor visibility.

When comparing the initial BoNT-A injection dose-response in the right arm (96-week study) to the left arm (current study), the decrease in tremor amplitude measured by kinematics and visual rating (FTM part A) show similar reduction in severity. Likewise, the decrease in maximal grip strength in the left arm, which majority of participants did not perceive as bothersome, and the reported non-debilitating finger weakness was expectantly like the initial weakness response experienced by participants in the right arm (Figure 2B–D) [17]. The ET and PD participants tolerability to additional muscles treated with BoNT-A for bilateral arm treatment did not impact their muscle weakness profile.

When comparing the tremor characteristics for each participant between the left and right arm, no global pattern in arm position or tasks was to be found. Furthermore, tremor at the wrist, elbow, and shoulder for each arm was unique for participants (Table 2). Thus, for optimal BoNT-A injection parameter customization, several arm positions or tasks must be conducted as tremor severity is variable depending on arm positioning. The individualized wrist, elbow, and shoulder dosing for muscles in each arm requires a high level of customization which is feasible by objective tremor analysis and is overwhelming and time consuming when done by visual (clinical) assessment (Supplemental Tables S1–S4). When comparing the left and right arm initial injection dosage within each participant, no obvious dose pattern was present for individual muscle targets; for the wrist specifically, some muscles were not treated if the tremor analysis showed minimal muscle group involvement for one arm but was present in the other arm (Supplemental Tables S3 and S4).

Given this was a small case series on select participants with asymmetric bilateral arm tremor (worse tremor in the right) and who were not naïve to BoNT-A, the trend suggests the use of bilateral tremor therapy provides greater benefit in QoL for those with worsening or equally severe tremor on both sides. It would be interesting to explore the use of bilateral BoNT-A therapy in a larger cohort of both BoNT-A-naïve ET and PD patients, and for these individuals to present with equally bothersome tremor on both sides to compare efficacy and tolerability outcomes to our present study. The findings from such a study would also be interesting to compare to the previous unilateral study to see if QoL and functional improvements are superior with fewer treatment cycles required and how patients perceive their arm weakness if treatment in both arms were initiated jointly. Presently, the overall combined dose for both arms ranged from 120–450 U and none of the 7 tremor participants experienced bothersome muscle weakness side-effects during bilateral treatment. One of the other limitations in this current report is the 18 weeks of available data; it would be of significant real-world value to conduct a longer study to see if the QoL and functional benefits witnessed are maintained following the first two treatment cycles in both arms.

Ultimately, this pilot bilateral tremor study gives us the first glimpse of the opportunity for treating patients with BoNT-A aided by tremor analysis. Presence of bilateral tremor alone might not necessitate the need to treat both sides, but instead require careful clinical consideration of objective tremor severity, handedness, and of course the patient’s expectation of outcome; whether its visible suppression of tremor, or their desire for improvement in quality of life and hand function when performing bimanual tasks.

## 4. Materials and Methods 

### 4.1. Study Design and Participants

An extension of a previously published single-centre, single-injector, open-label, clinical phase II pilot study by the London Movement Disorders Centre [15,16,17] was approved by the Western University Health Sciences Research Ethics Board (REB) on 9 September 2015, the REB study number is: 101749. Participants who received benefit in the original 96-weeks [17], continued to receive unilateral BoNT-A therapy and were identified to have bilateral tremor with bothersome tremor present on the untreated side. For the ET participants, they had presentation of bilateral tremor at the start of the unilateral study [17], however, majority of these ET participants had significantly more severe tremor in their dominant right hand, which received unilateral therapy, as compared to their left hand. These participants were given the choice to start receiving injections in their other upper (left) limb if participants perceived their tremor to be bothersome/disabling. 7 out of 21 participants (5 ET and 2 PD participants) decided to receive BoNT-A injection therapy in their other upper (left) limb. This report described a total of four study visits every 6 weeks totaling 18-weeks where participants received injections 12-weeks apart at transition visits 1 (T1) and 3 (T3) and follow-ups were conducted 6-weeks post-injection at transition visits 2 (T2) and 4 (T4). First participant’s first visit and last participant’s last visit occurred between August 2016 and May 2018. All participants provided written informed consent. The study and all related trials for this intervention are registered on the clinicaltrials.gov registry (ClinicalTrials.gov Identifier: NCT02427646).

### 4.2. Clinical Outcome Measures and Kinematic Tremor Analysis

Severity of tremor and limb functionality were reported using the Fahn-Tolosa-Marin (FTM) tremor rating scale, quality of life (QoL) measures were reported using the quality of life for essential tremor questionnaire (QUEST) [18,19]. Muscle weakness as a side effect to BoNT-A injections was monitored using a Baseline^®^ hydraulic hand dynamometer (Item#: 12-0240, White Plains, NY, USA) to measure maximal grip strength [20]. A Likert style participant-rated scale ranging from 0: no weakness, 1: mild, 2: moderate, 3: marked and 4: severe weakness with functional loss in injected muscles was administered to assess perceived muscle weakness reported by all participants at each visit. In addition, manual muscle testing (MMT) was used to assess strength in the fingers, wrist, and elbow flexor/extensor muscles where a score of 3+ or higher representing the ability to hold muscle position against gravity and resist slight pressure [21].

Severity of upper limb tremor was captured with kinematic sensors while participants performed a series of six scripted tasks each held for 20 s over three trials, as previously described [15]. Wrist tremor was analyzed in multiple degrees of freedom (DOFs) which resulted in the decomposition of tremor muscle groups such as wrist flexion-extension (F/E), radial-ulnar (R/U), and pronation-supination (P/S). Kinematics was utilized to monitor change in tremor severity over the 18-week study. All participants were assessed while “ON” their medication and during the same time of day to reduce tremor variability.

### 4.3. Treatment

Participants received two treatments 12 weeks apart, bilateral, intramuscular injections of BoNT-A (incobotulinumtoxinA; Xeomin^®^, Merz Pharmaceuticals, Frankfurt, Germany) of a total dose ranging between 30–300 U into muscles of the wrist, elbow and/or shoulder. Injection parameters previously used for the originally treated right arm (injector’s experience with visual and kinematic guidance to allocate dosages based on highest total tremor amplitude at each arm joint) were optimized for several cycles prior to the patient’s transition to bilateral therapy [17]. For the newly treated left arm, kinematic analysis was conducted to determine the initial injection parameters for the transition arm by identifying the task that produced the highest tremor amplitude per arm joint. The initial BoNT-A dose per muscle for the left arm was based on kinematic tremor analysis; a dosing algorithm was created based on the relationship of total joint dose and highest joint tremor severity from the earlier unilateral tremor-toxin study [17]. Dosages per muscle for the second treatment cycle were adjusted by the injector based on changes in kinematic tremor severity from pre- to post-injection time-points, clinical judgement and with patient feedback, such as noticeable muscle weakness as previously described by Samotus et al. [17].

### 4.4. Analysis

No formal sample size calculation was performed, all analysis was exploratory, and clinical and kinematic data are presented as case reports. Mean clinical scale scores and RMS tremor amplitudes from the ET and PD population groups were plotted for all transition study visits (T1–T4). Changes in mean clinical scores and RMS tremor amplitudes, as mean percent change and mean point/RMS degree changes, were compared from baseline (T1) to each post-treatment time-point (week 6 (T2), 12 (T3) and 18 (T4)), between re-injection visits (baseline (T1) and week 12 (T3)) and between peak effect of BoNT-A (weeks 6 (T2) and 18 (T4)). FTM part A scores from weeks 0 (L1) and 96 (L13) were plotted from data previously published in Samotus et al. to report any changes in tremor severity prior to initiation of transition treatment [17].

## 5. Patents

Two patents, PCT/CA2013/000804 pending to MDDT Inc. (London, ON, Canada), and a patent PCT/CA2014/050893 pending to MDDT Inc., resulted from the work reported in this manuscript.

## Figures and Tables

**Table 1:** Participant demographics and injection dosages, number of muscles and number of treatment cycles administered in the optimized, original treated right arm prior to transition into bilateral therapy.

ID	Diagnosis	Gender	Age	Medications (Daily Dose)	Dominant Limb	First Injected Limb	7th Injection–96 Weeks		
							Total Dose (Units)	# of Muscles Treated	# of Additional Unilateral Injections after 96-Weeks but before Transition
1	PD	M	35	Stalevo (400 mg)	R	R	300	13	10
2	ET	F	74	Primidone (125 mg)	R	R	85	7	8
3	ET	M	78	Primidone (125 mg)	R	R	200	8	9
4	PD	M	68	Sinemet (750 mg)	R	R	200	11	7
5	ET	F	65	-	R	R	280	13	6
6	ET	F	80	-	R	R	165	11	2
7	ET	M	73	-	R	R	115	9	3

Number (#).

**Table 2:** The task with the highest tremor amplitude observed at each arm joint in the newly treated arm (left) and in the original treated arm (right).

ID	Diagnosis	Task with Highest Tremor Amplitude in the “Newly Treated Arm” (Left Arm)			Task with Highest Tremor Amplitude in the “Original Treated Arm” (Right Arm) *		
		Wrist	Elbow	Shoulder	Wrist	Elbow	Shoulder
1	PD	Posture-1	Posture-2	Posture-2	Load-1	Posture-2	Load-1
2	ET	Posture-1	Posture-2	Posture-2	Load-2	Load-2	Posture-2
3	ET	Load-1	Load-2	Load-2	Load-1	Load-2	Load-2
4	PD	Posture-2	Posture-2	Posture-1	Load-1	Load-2	Load-2
5	ET	Load-2	Load-1	Load-1	Rest-2	Posture-1	Load-2
6	ET	Posture-1	Load-2	Load-2	Load-1	Load-2	Load-2
7	ET	Posture-1	Posture-1	Rest-2	Posture-2	Load-1	Posture-2

* Tremor analysis of the original treated arm was performed during the conduct of the previously published report by Samotus O. et al., 2017.

## Supplementary Materials

The following are available online http://www.mdpi.com/2072-6651/10/10/394/s1, Table S1: Optimization of Botulinum toxin type A (BoNT-A) parameters over the two injection treatments in the optimized, original treated limb (right arm) (A) and in the newly treated limb (left arm) (B), Table S2: Optimization of BoNT-A parameters over the two injection treatments in the newly treated limb (left arm), Table S3: The muscles and BoNT-A dosing parameters over the two injection treatments in the original treated limb (right arm), Table S4: The muscles and BoNT-A dosing parameters over the two injection treatments in the newly treated limb (left arm).

## Abbreviations

BoNT-A	botulinum toxin type A
DOF	degree of freedom
ET	essential tremor
FTM	Fahn-Tolosa-Marin
MMT	manual muscle testing
PD	Parkinson’s disease
QoL	quality of life
QUEST	Quality of Life in Essential Tremor Questionnaire
RMS	root mean square
