# Supplementary material for: Transitioning from Unilateral to Bilateral Upper Limb Tremor Therapy for Parkinson’s Disease and Essential Tremor Using Botulinum Toxin: Case Series
Source: Toxins (Basel). 2018 Sep 27;10(10):394. doi: 10.3390/toxins10100394 (PMC6215170; doi:10.3390/toxins10100394)
Supplement: Supplementary file 1 [file toxins-10-00394-s001.pdf]

Supplementary Materials: Transitioning from Unilateral to Bilateral Upper Limb Tremor Therapy for Parkinson's Disease and Essential Tremor Using Botulinum Toxin: Case Series

Olivia Samotus, Jack Lee and Mandar Jog

Table S1. Optimization of botulinum toxin type A (BoNT-A) parameters over the two injection treatments in the optimized, original treated limb (right arm) (A) and in the newly treated limb (left arm) (B).

ID	First Transition Injection Cycle (T1) in Original Treated (Right) Limb					Second Transition Injection Cycle (T3) in Original Treated (Right) Limb				
	Wrist Dose	Elbow Dose	Shoulder Dose	Total Dose (U)	# of Muscles	Wrist Dose	Elbow Dose	Shoulder Dose	Total Dose (U)	# of Muscles
1	75	50	100	225	13	75	50	100	225	13
2	25	10	25	60	7	25	10	25	60	7
3	145	40	0	185	8	145	35	0	180	8
4	70	35	50	155	11	60	35	50	145	11
5	60	60	90	210	12	60	60	90	210	12
6	70	45	35	150	11	70	45	35	150	11
7	60	40	0	100	9	55	40	0	95	9
Mean	72.1	40.0	42.9	155.0	10.1	70.0	39.3	42.9	152.1	10.1
SD	36.2	15.5	40.0	59.0	2.2	36.7	15.7	40.0	59.5	2.2

Number (#).

Table S2. Optimization of BoNT-A parameters over the two injection treatments in the newly treated limb (left arm).

ID	First Transition Injection Cycle (T1) in Newly Treated (Left) Limb					Second Transition Injection Cycle (T3) in Newly Treated (Left) Limb				
	Wrist Dose	Elbow Dose	Shoulder Dose	Total Dose (U)	# of Muscles	Wrist Dose	Elbow Dose	Shoulder Dose	Total Dose (U)	# of Muscles
1	65	60	75	200	11	65	60	75	200	11
2	30	30	35	95	10	25	30	35	90	10
3	40	30	35	105	12	40	40	35	115	12
4	55	40	40	135	11	55	50	40	145	11
5	25	30	40	95	10	25	30	40	95	10
6	70	50	80	200	12	70	50	80	200	12
7	40	30	35	105	10	35	30	35	100	10
Mean	46.4	38.6	48.6	133.6	10.9	45.0	41.4	48.6	135.0	10.9
SD	17.3	12.1	19.9	47.3	0.9	18.5	12.1	19.9	48.0	0.9

Number (#).

Table S3. The muscles and BoNT-A dosing parameters over the two injection treatments in the original treated limb (right arm).

	ID	1	2	3	4	5	6	7	Mean	SD	Range (Min)	Range (Max)
**First Transition Injection Cycle (T1) in Original Treated (Right) Limb**	**Flexor carpi radialis**	5	5	15	20	5	10	5	9.3	6.1	5	20
	**Flexor carpi ulnaris**	5	5	15	20	10	10	5	10.0	5.8	5	20
	**Extensor carpi radialis**	10		15	5	10	10	5	9.2	3.8	5	15
	**Extensor carpi ulnaris**	10		15	5	15	10	5	10.0	4.5	5	15
	**Pronator teres**	15	5	30	5	10	10	15	12.9	8.6	5	30
	**Pronator quadratus**	15	5	30	5	10	10	15	12.9	8.6	5	30
	**Supinator**	15	5	25	10		10	10	12.5	6.9	5	25
	**Biceps**	20	10	40	15	30	15	20	21.4	10.3	10	40
	**Triceps**	30			20	30	30	20	26.0	5.5	20	30
	**Pectoris major**	25	25		25	25	20		24.0	2.2	20	25
	**Teres major**	25				25	15		21.7	5.8	15	25
	**Deltoid**	25				20			22.5	3.5	20	25
	**Supraspinatus**	25			25	20			23.3	2.9	20	25
**Second Transition Injection Cycle (T3) in Original Treated (Right) Limb**	**Flexor carpi radialis**	5	5	15	15	5	10	5	8.6	4.8	5	15
	**Flexor carpi ulnaris**	5	5	15	15	10	10	5	9.3	4.5	5	15
	**Extensor carpi radialis**	10		15	5	10	10	5	9.2	3.8	5	15
	**Extensor carpi ulnaris**	10		15	5	15	10	5	10.0	4.5	5	15
	**Pronator teres**	15	5	30	5	10	10	15	12.9	8.6	5	30
	**Pronator quadratus**	15	5	30	5	10	10	15	12.9	8.6	5	30
	**Supinator**	15	5	25	10		10	5	11.7	7.5	5	25
	**Biceps**	20	10	35	15	30	15	20	20.7	8.9	10	35
	**Triceps**	30			20	30	30	20	26.0	5.5	20	30
	**Pectoris major**	25	25		25	25	20		24.0	2.2	20	25
	**Teres major**	25				25	15		21.7	5.8	15	25
	**Deltoid**	25				20			22.5	3.5	20	25
	**Supraspinatus**	25			25	20			23.3	2.9	20	25

Table S4. The muscles and BoNT-A dosing parameters over the two injection treatments in the newly treated limb (left arm).

	ID	1	2	3	4	5	6	7	Mean	SD	Range (Min)	Range (Max)
**First Transition Injection Cycle (T1) in newly treated (left) limb**	**Flexor carpi radialis**	15	5	5	10		10	10	9.2	3.8	5	15
	**Flexor carpi ulnaris**	15		5	5		10	5	8.0	4.5	5	15
	**Extensor carpi radialis**	15	10	5	20	5	20	10	12.1	6.4	5	20
	**Extensor carpi ulnaris**	15	5	5	15	5	20	5	10.0	6.5	5	20
	**Pronator teres**		5	5		5	5		5.0	0.0	5	5
	**Pronator quadratus**		5	5		5	5		5.0	0.0	5	5
	**Supinator**	5		10	5	5		10	7.0	2.7	5	10
	**Biceps**	30	15	15	20	15	25	15	19.3	6.1	15	30
	**Triceps**	30	15	15	20	15	25	15	19.3	6.1	15	30
	**Pectoris major**	40	20	20	15	20	40	20	25.0	10.4	15	40
	**Teres major**	15	5	5	5	10	10	5	7.9	3.9	5	15
	**Deltoid**	10			10		15		11.7	2.9	10	15
	**Supraspinatus**	10	10	10	10	10	15	10	10.7	1.9	10	15
**Second Transition Injection Cycle (T3) in newly treated (left) limb**	**Flexor carpi radialis**	15	5	5	10		10	10	9.2	3.8	5	15
	**Flexor carpi ulnaris**	15		5	5		10	5	8.0	4.5	5	15
	**Extensor carpi radialis**	15	5	5	20	5	20	10	11.4	6.9	5	20
	**Extensor carpi ulnaris**	15	5	5	15	5	20	5	10.0	6.5	5	20
	**Pronator teres**		5	5		5	5		5.0	0.0	5	5
	**Pronator quadratus**		5	5		5	5		5.0	0.0	5	5
	**Supinator**	5		10	5	5		5	6.0	2.2	5	10
	**Biceps**	30	15	20	25	15	25	15	20.7	6.1	15	30
	**Triceps**	30	15	20	25	15	25	15	20.7	6.1	15	30
	**Pectoris major**	40	20	20	15	20	40	20	25.0	10.4	15	40
	**Teres major**	15	5	5	5	10	10	5	7.9	3.9	5	15
	**Deltoid**	10			10		15		11.7	2.9	10	15
	**Supraspinatus**	10	10	10	10	10	15	10	10.7	1.9	10	15
